# Shakespeare—A New Discovery

**Published:** 1989-08

**Authors:** Martin Crosfill

**Affiliations:** Consultant Surgeon. West Cornwall Hospital, Penzance


### SHAKESPEARE — A new discovery

**Martin Crosfill**
Consultant Surgeon. West Cornwall Hospital, Penzance

It is a rare event when a previously unknown manuscript by a classical author is discovered. The provenance of the fragment here reproduced is unknown, its authenticity is beyond question if for no other reason than that the folio is signed on verso by Shakespeare himself, using three different inks, three styles of script and eight spellings.

Shakespeare's knowledge of medicine has been extensively documented[1–3] but the dramatic episode presented here was previously unrecorded and no play on the subject is known to have been performed. Probably the idea was discarded because the text has been extensively reworked into later productions. A great deal of research has gone into tracing the later echoes of this admittedly immature work and references are appended.

It is well known that Shakespeare's son-in-law was a GP but this episode takes place in hospital. On the assumption that the author had already moved to London, the hospital background can only have been provided by St. Thomas' or Barts. Internal evidence, in particular the grotesquely distorted caricature of consultant behaviour suggests that the viewpoint was that of a member of the junior medical staff. It is quite possible that his informant was Dr Rodrigo Lopez, first House Physician to Barts., who was hanged, drawn and quartered in 1594 though not, apparently, for slandering the consultant staff.

| | |
|---|---|
| SCENE: | A hospital ward<br>Enter a consultant and House Physician. |
| CONS: | Good morrow fairest sister[4] |
| SISTER: | (checking the time) Here, I take it, is the doctor come.[5] |
| CONS: | Find you out a bed.[6] |
| SISTER: | There's never a one,[7] and for three months before, no interim, not a minute's vacancy both day and night.[8] |
| CONS: | Make space enough between[9] |
| STAFF NURSE: | Are there not men in your ward sufficient?[10] |
| SISTER | My staff understands me.[11] How does your patient, doctor?[12] |
| CONS: | He is very sick and would to bed.[13] |
| PATIENT: | I am very sick.[14] |
| CONS: | My bed shall be abused,[15] I will discharge thee.[16] You hie you home to bed.[17] See him presently discharged.[18] |
| PATIENT: | First I began in private with you.[19] |
| CONS: | That be all the difference.[20] (to H.P.) Pray you, examine him.[21] |
| H. P.: | Sirrah! I must examine thee.[22] |
| PATIENT: | Were you the doctor and I knew you not?[23]<br>O excellent young man![24] |
| H.P.: | Where lies thy pain?[25] |
| PATIENT: | Sit thou by my bed and hear.[26] I have a pain upon my forehead here[27]—Lord how my head aches, what a head have I![28] |
| Note | There are no stage directions on the original document. For clarity these have been added. |
| H. P.: | How, art thou out of breath? |
| PATIENT: | Do you not see that I am out of breath?[28] |
| H. P.: | How art thou out of breath when thou hast breath to say to me that thou art out of breath? Give me your hand and let me feel your pulse.[29] |
| CONS: | You go not the way to examine[30] (picks up chart). Through this distemper we see that rheumatic diseases do abound.[31] Your hand, your tongue[32]—foul wind is but foul breath and foul breath is noisesome![33]<br>Sits the wind in that corner? (palpates)[34]<br>Stop in your wind sir![35]<br>What heard you[36] |
| H. P.: | I heard nothing[37] |
| CONS: | Teach your ears to list me with more heed[38] |
| H. P.: | (tries again) Upon mine honour sir, I heard a humming.[37] |
| CONS: | (listens to the chest) . . . the current that with gentle murmur glides, thou knowest, being stopp'd impatiently doth rage but when his fair course is not hindered he makes sweet music.[39]<br>Thy heart is big, get thee apart and weep.[40] |
| PATIENT: | Let me not die your debtor[41] |
| STAFF NURSE: | The doctor is well moneyed.[42] |
| CONS: | I have seen a medicine that's able to breathe life into a stone.[43]<br>Fetch me this herb.[44] |
| SISTER: | I am the drudge and toil in your delight but you shall bear the burden soon.[45]<br>Hence forth do your messages yourself. We have no staff.[46] I . . . make the beds and do all myself.[47] |
| CONS: | See how my wretched sister sobs.[48] Get you some of this distilled Carduus Benedictus and lay it to your heart. It is the only thing for a qualm.[49] |
| H. P.: | I'll fetch it for you[50] |
| PATIENT: | (panicking) Come, bring me to some private place.[51] Let me have surgeons.[52] |
| CONS: | Lets take leave of him.[53] Do not interrupt me in my course.[54] You are the man must stead us all.[55] I shall be sent for in private to him.[56]<br>(exit with House Physician) |
| H. P.: | (sotto voce) Cans't thou bring me to the party?[57] I have yet room for six scotches.[58] |
| STAFF NURSE: | (to no one in particular) I'll have that doctor for my bedfellow.[59] |

### REFERENCES

[1] Shakespeare and Medicine. Simpson R. R. Livingstone 1959

[2] St. Mary's Hosp. Gazette. Black H. December 1933 pp 30–40
[3] J. Roy. Naval Med. Service. Crosfill J. W. L. 1952 *XXXVII* p114 et seg.
[4] Cymbeline II 3
[5] Merchant of V. IV 1
[6] M. N. D II 2
[7] Timon V 1
[8] Twelfth Night V 1
[9] Antony & Cleo. II 3
[10] Measure for Meas. II 1
[11] Two Gentleman II 5
[12] Macbeth V 3
[13] Henry V II 1
[14] Merchant of V. IV 1
[15] Merry Wives II 2
[16] Tempest I 2
[17] Two Gentlemen IV 2
[18] Com. Errors IV 4
[19] Henry VIII II 4
[20] Two Gentlemen IV 4
[21] Much Ado V 1
[22] 2 Henry VI IV 2
[23] Merchant of V. V 1
[24] Merchant of V. IV 1
[25] L.L.L. IV 3
[26] 2 Henry IV IV 5
[27] Othello III 3
[28] Rom & Jul II 5
[29] Com. Errors IV 4
[30] Much Ado IV 2
[31] M. N. D. II 1
[32] Macbeth I 5
[33] Much Ado V 2
[34] Ibid II 3
[35] Com. Errors I 2
[36] Much Ado IV 2
[37] Tempest II 1
[38] Com. Errors IV 1
[39] Two Gentlemen II 7
[40] Jul Caesar III 1
[41] L. L. L. V 2
[42] Merry Wives IV 4
[43] Alls Well II 1
[44] M. N. D. II 1
[45] Rom & Jul. II 5
[46] 3 Henry VI II 1
[47] Merry Wives I 4
[48] Titus III 1
[49] Much Ado III 4
[50] Merry Wives I 4
[51] Pericles IV 6
[52] Lear IV 6
[53] Tempest I 1
[54] Rom & Jul V 3
[55] Taming of S. I 2
[56] 2 Henry IV V 5
[57] Tempest III 2
[58] Anthony & Cleo. IV 7
[59] Merchant of V. V 1

